# Using complex networks for refining survival prognosis in prostate cancer patient

**DOI:** 10.12688/f1000research.8282.1

**Published:** 2016-11-16

**Authors:** Massimiliano Zanin

**Affiliations:** 1 Innaxis Foundation & Research Institute, Madrid, Spain; 2 Department of Electrical Engineering, Faculty of Sciences and Technology, Universidade Nova de Lisboa, Caparica, Portugal

**Keywords:** Prostate cancer, survival prognosis, complex networks, classification

## Abstract

Complex network theory has been used, during the last decade, to understand the structures behind complex biological problems, yielding new knowledge in a large number of situations. Nevertheless, such knowledge has remained mostly qualitative. In this contribution, I show how information extracted from a network representation can be used in a quantitative way, to improve the score of a classification task. As a test bed, I consider a dataset corresponding to patients suffering from prostate cancer, and the task of successfully prognosing their survival. When information from a complex network representation is added on top of a simple classification model, the error is reduced from 27.9% to 23.8%. This confirms that network theory can be used to synthesize information that may not readily be accessible by standard data mining algorithms.

## Introduction

Constructing prognostic models for different types of cancers is a problem that is attracting increasing attention, due to the high impact that these models may have in the clinical treatment. This is clearly related to the movement of personalized medicine ( Jain, 2005; Samani *et al.*, 2010; Van't Veer & Bernards, 2008). As more and more data describing human biology are available, both for healthy and pathological conditions, coming from heterogeneous sources ( *e.g.* from all the -omics fields), there is a well-founded hope that such data may be of help to improve the treatment of individual patients, personalizing the way drugs and therapies are provided.

When one ought to extract a model from a collection of data, the customary solution is to resort to *data mining* algorithms. In the case of cancer prognosis, this has resulted in the development of numerous models - see, for instance, Alexe *et al.*, 2006; Gupta *et al.*, 2011; Halabi *et al.*, 2003; Halabi *et al.*, 2014; Mangasarian & Wolberg, 2000 and Quaranta *et al.*, 2005 for a few examples. Data mining nevertheless presents some drawbacks, the most important of which is the way features are analyzed. Elements are considered individually, or by being pairwise combined; yet, data mining does not provide a way to create a global picture of the available data.

In the last decade, a novel solution has been proposed. The *complex network theory* provides an elegant way for representing the structure created by the interactions between the elements of a complex system ( Boccaletti *et al.*, 2006; Strogatz, 2001). The result is encoded in an *adjacency matrix*, which can then be analyzed by means of multiple metrics ( Costa *et al.*, 2007). Applications span from the characterization of social networks, to the internet or the human brain ( Costa *et al.*, 2011).

In this contribution, I explore the possibility of using complex networks as an instrument for improving a model of survival prognosis of patients with metastatic castrate resistant prostate cancer (mCRPC) treated with docetaxel. In order to achieve this, I compare two models. The first one is a classification model, *i.e.* classifying between surviving and non-surviving patients, which only uses raw features like baseline lab results and patient vital signs. The second one combines such information with structural metrics extracted from a network representation of the same data. The hypothesis tested here is that complex networks should synthesize information present in the raw data in a new way that should reflect an improved classification score (Zanin *et al.*, 2014b).

The paper is organized as follows: first, I describe the main methods of the analysis, with a special focus on the networks reconstruction methodology and the metrics used for their characterization, and the dataset considered here; afterwards, the results obtained are presented, *i.e.* the comparison of the two classification models; finally, some conclusions are drawn.

## Methods

### Network reconstruction

Reconstructing a network representation of a given system entails two steps. First, one needs to define the elements of such a system. This is usually constrained by the type of available data; thus, in this case, the nodes of the network are going to correspond to the different available biomarkers.

Second, one should detect when two of such elements are connected by some kind of relationship. If *a priori* knowledge is available, *e.g.* information about how different metabolites or proteins are connected in a pathway, such information can directly be mapped into the network. Alternatively, if a time evolution ( *i.e.* a time series) is available for each element, *functional* links can be established between them, by means of metrics like correlations or causalities. Note that this last option entails two important problems: a time evolution should be available, which is not straightforward in the case of biomedical analyses; and that functional links represent the “co-evolution” of factors, while in some cases, and specifically in the diagnosis of a disease, it is more interesting to detect “deviations” from the expected (healthy) behavior.

Recently, a new methodology for network reconstruction has been proposed, which solves the two aforementioned problems ( Zanin & Boccaletti, 2011; Zanin *et al.*, 2014). Starting with a set of scalar values, pairs of elements are analyzed by firstly detecting if a standard relation is present between them in a set of control subjects; afterwards, data corresponding to new subjects are compared with such relation, and a link is created between two nodes if they present an *abnormal* deviation. The resulting object is called a *parenclitic network*, named after the Greek term for “deviation”, originally used by the Greek philosopher Epicurus to designate the spontaneous and unpredictable swerving of free-falling atoms ( Zanin *et al.*, 2014).

In mathematical terms, suppose *n* healthy subjects are described by a vector of features, such that the *i*-th of them is represented by *f _i_* = ( *f*_*i,*1_, *f*_*i,*2_, … , *f _i,n _f__*). All the *n _f_* features are mapped into nodes of the network, which is now described by an adjacency matrix *A _n _f_×n _f__*. As the final aim is to construct a network for each subject under study, suppose a new subject *j*, with its corresponding vector *f _j_*, is introduced in the system. The reconstruction process should analyze each pair of features, denoted by *k* and *l*, to understand if they deviated from the expected (healthy) behavior. For the sake of simplicity, in this work we consider that the healthy relation can be obtained as a linear regression between both features:

*f. _,l_* = *α _k,l_* + *β _k,l_f. _,k_* + *∈ _k,l_.*

Here, *f. _,k_* represents the vector of values of feature *k* for all healthy subjects, and *α* *_k,l_* and *β* *_k,l_* the two parameters of the best linear fit. Additionally, *∈ _k,l_* is a vector containing all fit errors; note that a linear relation may not describe well the relationship between *k* and *l*, and that this vector will be key to understand its statistical significance. Now, suppose a new subject *h* is available, for which their health condition is unknown, and for which one wants to create the corresponding network representation. A link between nodes *k* and *l* is then created, with a weight equal to its distance from the previously detected normal relation:

$$w_{k,l}=\frac{f_{h,l}–\left(\alpha_{k_{,}l}+\beta_{k_{,}l}f_{h,k}\right)}{\sigma_{k,l}},$$

being *σ _k,l_* the standard deviation of *∈ _k,l_*. In other words, *w _k,l_* represents the Z-score of the distance of the subject *h* with respect to the normal behavior of features *k* and *l* - large values of *w _k,l_*, both positive and negative, indicate that the subject under analysis presents an abnormal behavior, which may be symptomatic of a disease. When the process is repeated for all pairs of features, the result is a *parenclitic network* for each patient.

### Network interpretation

Intuitively, healthy subjects should be associated with random-like networks, as strong links may appear due to the intrinsic noise of biological processes, but should not form coherent structures; on the other hand, patients should present networks with non-trivial topologies. Also, the more a network is different from a random structure, the more severe the pathology is expected to be.

In order to transform the obtained networks into a representation suitable to be used in a data mining (classification) algorithm, first these have been binarized, *i.e.* links with a weight | *w _k,l_*| ≤ 0.5 have been discarded. The threshold of 0.5 has manually been set, in order to obtain structures dense enough to support the subsequent analysis, but still being able to discard statistically insignificant connections. Afterwards, two topological ( *i.e.* structural) properties have been considered:

- *Link density*, defined as the number of links present in the network, divided by the number of all possible links. The higher the link density, the more pairs of features present an abnormal behavior.
- *Information content* ( Zanin *et al.*, 2014). This metric assesses the presence of mesoscale structures, *i.e.* structures created by small groups of nodes, by evaluating the information lost when pairs of nodes are iteratively merged together. Low values of Information Content indicate a random-like structure; conversely, high values suggest a non-trivial topology, potentially fingerprint of a severe condition.

### Classification

In order to evaluate the performance of a complex network representation with respect to a baseline, a classification between the two groups of patients ( *i.e.* surviving *vs.* not surviving patients) is performed, and the resulting scores compared. Such classification is based on a support vector machine (SVM) model with linear kernel ( Noble, 2006; Wang, 2005).

SVMs are binary linear classifiers that model concepts by creating hyperplanes in a multidimensional space, which can be used for both classification and regression ( Cortes & Vapnik, 1995). A good separation is achieved by the hyperplane that has the largest distance to the nearest training-data point of any class, as this minimises the error. The SVM model has been chosen for two reasons: its good performance and diffusion in biomedical classification problems; and its simplicity: only linear relationships are mined, allowing a better identification of the contribution of the complex network representation.

The validation of the results has been performed using a 10-fold cross-validation ( Friedman *et al.*, 2001). The original sample of subjects is randomly partitioned into 10 equal sized subsamples. A single subsample is retained as the validation data for testing the model, and the remaining 9 subsamples are used as training data. The cross-validation process is then repeated 10 times, with each of the 10 subsamples used exactly once as the validation data. The average value of the error obtained in the 10 executions is used for estimating the error.

### Initial dataset

The dataset considered here is part of the Prostate Cancer DREAM Challenge, including information from the prostate cancer clinical trials ASCENT-2 (Novacea, provided by Memorial Sloan Kettering Cancer Center) ( Scher *et al.*, 2011), VENICE (Sanofi) ( Tannock *et al.*, 2013), MAINSAIL (Celgene) ( Petrylak *et al.*, 2015), and ENTHUSE-33 (AstraZeneca) ( Fizazi *et al.*, 2013). Only the data included in the CoreTable have been considered, representing the core patient level data. They cover information about demographics, co-existing disease conditions, prior treatment of the tumor and other co-existing conditions, important baseline lab results and vital signs, lesion measure and early response to therapy. More information on the dataset can be found at https://www.synpase.org/ProstateCancerChallenge.

One of the limitations of the network reconstruction process previously described is that it can only handle numerical features. Thus, only those features fulfilling this condition have been selected. Additionally, binary features have been transformed into numbers, *i.e.* 1 for “yes” and 0 for “no”. The final data set included 92 features for each patient.

Afterwards, 2000 patients have been randomly selected, of which half of them did not survive cancer - as coded by the DEATH flag in the dataset. The rationale of selecting only a subset of patients is two-fold: first, to reduce the computational cost, and thus allow a more detailed analysis of results; and second, to ensure that the data set used in the classification task is balanced, *i.e.* it includes the same number of subjects in both classes. All other patients have been discarded.

## Results

### Standard scenario: raw features classification

**Figure 1.**
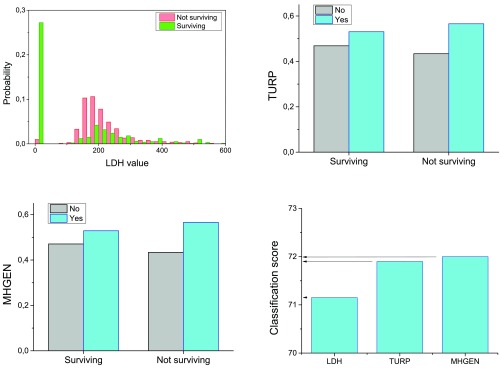
Classification with raw features. Probability distributions of the LDH feature for surviving and not surviving patients (top left). Appearance probability of the features TURP and MHGEN, for surviving and not surviving patients (top right and bottom left). Classification score when considering LDH, LDH + TURP, and all three features (bottom right).

Figure 1 presents the results obtained in the classification of patients using only raw features. As previously introduced, this classification will be the baseline against which the benefits of using complex networks will be evaluated. In order to reduce the computational cost of the analysis, and to reduce the risk of overfitting, a greedy feature selection algorithm has been executed. The three selected features were: LDH ( *Lactate Dehydrogenase* level), TURP (prior transurethral resection of the prostate, binary value) and MHGEN (presence of general disorders, binary value). The probability distributions for the three features are presented in Figure 1 top and bottom left.

By using these three selected features, the classification score reaches 72.1% ( Figure 1, bottom right). Adding more features does not yield substantial improvements.

### Enhanced scenario: complex network features

In the second case, I consider the same original raw features, plus the two features synthesized from the complex network representation, as previously described. A network has been created for each subject, by using the information of surviving patients as baseline- in other words, surviving patients have been considered as *healthy*, following the convention previously described. In order to avoid overfitting, a new baseline has been calculated in each one of the 10 cross-validation rounds, ensuring no patient was included both in the training and in the classification steps. Finally, a greedy feature selection algorithm has been executed on the complete feature set, following the same process described previosuly.

**Figure 2.**
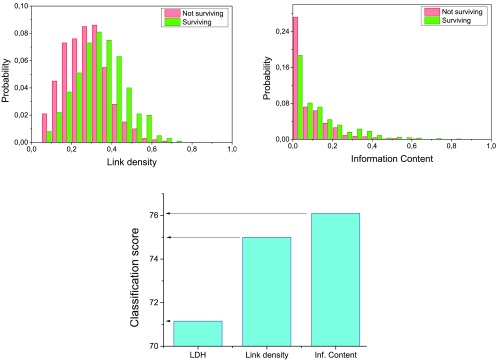
Classification with complex network features. (Top) Probability distributions of the link density and Information Content features, for surviving and not surviving patients. See main text for definitions. (Bottom) Classification score when considering LDH, LDH + link density, and all three features.

Figure 2 presents the results obtained, both in terms of the network features probability distributions (top), and the classification score (bottom). It can be appreciated as the classification score improves, from 72.1% up to 76.2%; this corresponds to a decrease of 15% in the classification error.

## Conclusions

If complex networks have by and large been used to *describe* biomedical problems ( Costa *et al.*, 2011), much less attention has been devoted to their relation with *prediction*, *i.e.* to how the information they provide could be used in the construction of diagnosis models. In this contribution, I make a first step in this direction, by studying the following hypothesis: can the precision of a predictive model be improved, if information extracted from a complex network representation is fed to a data mining algorithm along with raw features?

I used, as a test bed, a data set describing patients suffering from prostate cancer, and a classification task in which patients are discriminated according to the expected prognosis (surviving *vs.* not surviving). The inclusion of complex network features, obtained through a *parenclitic* representation ( Zanin & Boccaletti, 2011; Zanin *et al.*, 2014), resulted in a small but significant reduction of the classification error (from 27.9% to 23.8%).

When comparing these results with the state of the art, as for instance ( Halabi *et al.*, 2003; Halabi *et al.*, 2014), it is clear that they are still far away from representing an efficient prognostic instrument. Within the Prostate Cancer DREAM Challenge, the proposed method ranked 50 out of 51 in Subchallenge 1a (iAUC of 0.6171, against a reference of 0.7429 of the Halabi *et al.* method and 0.7915 of the winning team); and 27 out of 49 in Subchallenge 1b (RMSE of 214.39, against 194.41 of the winning team). Additionally, an error of the 23.8% in the survival probability is clearly intolerable for clinical applications.

It is also important to note that complex networks introduce a “black box” element in the analysis. As features are represented and analyzed in a topological way, *i.e.* focusing on the structure created by their relationships, it is not possible to identify which element(s) contribute the most to the final model. This complicates direct comparisons with standard prognostic models, and the design of therapeutic solutions.

In spite of the discussed drawbacks, we believe that the results here reported shed light on the importance of using complex networks in future prognostic models, as a way of synthesizing complex relationships in simple and numerical metrics.

## Data availability

The data referenced by this article are under copyright with the following copyright statement: Copyright: © 2016 Zanin M

Data associated with the article are available under the terms of the Creative Commons Zero "No rights reserved" data waiver (CC0 1.0 Public domain dedication). 

The Challenge datasets can be accessed at: https://www.projectdatasphere.org/projectdatasphere/html/pcdc

Challenge documentation, including the detailed description of the Challenge design, overall results, scoring scripts, and the clinical trials data dictionary can be found at: https://www.synapse.org/ProstateCancerChallenge

The code and documentation underlying the method presented in this paper can be found at: http://dx.doi.org/10.7303/syn4732239 ( Zanin, 2016)

## Supplementary material

Additional software, *e.g.* the MATLAB code for calculating the Information Content of a network, can also be found at: www.mzanin.com
