# Supplementary material for: Using complex networks for refining survival prognosis in prostate cancer patient
Source: F1000Res. 2016 Nov 16;5:2675. [Version 1] doi: 10.12688/f1000research.8282.1 (PMC5333606; doi:10.12688/f1000research.8282.1)
Supplement: Peer review history

# Peer review: Referee response for version 1

**DOI:** 10.5256/f1000research.8908.r18888

**Published:** 2017-02-27

**Authors:** Diego Raphael Amancio

**Affiliations:** 1 Institute of Mathematics and Computer Sciences, University of São Paulo, São Carlos, Brazil

The manuscript describes the application of complex networks concepts to the problem of identifying survival patients suffering from prostate cancer. The manuscript is scientifically sound, and the conclusions are supported by the results.

 The author, however, should address the following issues raised below: 

1. How performance depends on the weight chosen to binarize your data? Note that this step may affect considerably the performance.
2. Why traditional topological structures are not considered?
3. The author should further motivate the combination of traditional and network features, as similar approaches have been applied in other contexts ^1^^,^^2^.

I have read this submission. I believe that I have an appropriate level of expertise to confirm that it is of an acceptable scientific standard.

## References

[1] AmancioDR: A Complex Network Approach to Stylometry. *PLoS One*.2015;10(8) : 10.1371/journal.pone.0136076 e0136076 10.1371/journal.pone.0136076 26313921PMC4552030

[2] MotaNBFurtadoRMaiaPPCopelliMRibeiroS: Graph analysis of dream reports is especially informative about psychosis. *Sci Rep*.2014;4: 10.1038/srep03691 3691 10.1038/srep03691 24424108PMC3892182

# Peer review: Referee response for version 1

**DOI:** 10.5256/f1000research.8908.r17941

**Published:** 2016-11-24

**Authors:** Osvaldo Anibal Rosso

**Affiliations:** 1 Buenos Aires Institute of Technology (ITBA), Buenos Aires, Argentina

In the present manuscript the author explores the possibility of using complex networks as a tool for evaluation of the improving a model of survival prognosis of patients with metastatic castrate resistant prostate cancer treated with docetaxel. The main hypothesis tested in the manuscript is that complex networks should synthesize information present in raw data in a new way that should reflect an improved classification score, proposed by the author and co-workers previously ^1^.

 The manuscript is clearly and well written, and the conclusions are supported by the obtained results. I share the opinion of the author that the results of present research shed light on the importance of using complex networks in the future prognostic models. I recommend the indexing of the manuscript in the present form.

I have read this submission. I believe that I have an appropriate level of expertise to confirm that it is of an acceptable scientific standard.

## References

[1] ZaninMAlcazarJMCarbajosaJVPaezMGPapoDSousaPMenasalvasEBoccalettiS: Parenclitic networks: uncovering new functions in biological data. *Sci Rep*.2014;4: 10.1038/srep05112 5112 10.1038/srep05112 24870931PMC4037713
